# Stratified treatment of myocardial infarction with non-obstructive coronary arteries: the PROMISE trial

**DOI:** 10.1093/eurheartj/ehaf917

**Published:** 2025-10-28

**Authors:** Rocco A Montone, Nicola Cosentino, Riccardo Gorla, Simone Biscaglia, Giulia La Vecchia, Riccardo Rinaldi, Andrea Caffè, Marta Resta, Andrea Erriquez, Francesco Bedogni, Giampaolo Niccoli, Carlo Trani, Francesco Burzotta, Luca Testa, Federico De Marco, Filippo Crea, PROMISE Trial Investigators, Rocco A Montone, Giulia La Vecchia, Alice Bonanni, Andrea Caffè, Vincenzo Scarica, Antonio Maria Leone, Tommaso Sanna, Giovanna Liuzzo, Carlo Trani, Francesco Burzotta, Nicola Cosentino, Marta Resta, Claudia Lucci, Giancarlo Marenzi, Federico De Marco, Riccardo Gorla, Luca Testa, Francesco Bedogni, Simone Biscaglia, Gianluca Campo, Andrea Erriquez, Riccardo Rinaldi, Filippo Gurgoglione, Giampaolo Niccoli, Filippo Crea

**Affiliations:** Department of Cardiovascular Sciences, Fondazione Policlinico Universitario A. Gemelli IRCCS, L.go A. Gemelli, Rome 1–00168, Italy; Centro Cardiologico Monzino, IRCCS, Milan, Italy; Department of Clinical and Interventional Cardiology, IRCCS Policlinico, San Donato Milanese, Italy; Cardiology Unit, Azienda Ospedaliero Universitaria di Ferrara, Cona, Italy; Department of Cardiovascular and Pulmonary Sciences, Catholic University of the Sacred Heart, Rome, Italy; Center of Excellence in Cardiovascular Sciences, Isola Tiberina Hospital Gemelli Isola, Rome, Italy; Department of Cardiovascular and Pulmonary Sciences, Catholic University of the Sacred Heart, Rome, Italy; Department of Cardiovascular and Pulmonary Sciences, Catholic University of the Sacred Heart, Rome, Italy; Centro Cardiologico Monzino, IRCCS, Milan, Italy; Cardiology Unit, Azienda Ospedaliero Universitaria di Ferrara, Cona, Italy; Department of Clinical and Interventional Cardiology, IRCCS Policlinico, San Donato Milanese, Italy; Division of Cardiology, University of Parma, Parma University Hospital, Parma, Italy; Department of Cardiovascular Sciences, Fondazione Policlinico Universitario A. Gemelli IRCCS, L.go A. Gemelli, Rome 1–00168, Italy; Department of Cardiovascular and Pulmonary Sciences, Catholic University of the Sacred Heart, Rome, Italy; Department of Cardiovascular Sciences, Fondazione Policlinico Universitario A. Gemelli IRCCS, L.go A. Gemelli, Rome 1–00168, Italy; Department of Cardiovascular and Pulmonary Sciences, Catholic University of the Sacred Heart, Rome, Italy; Department of Clinical and Interventional Cardiology, IRCCS Policlinico, San Donato Milanese, Italy; Centro Cardiologico Monzino, IRCCS, Milan, Italy; Department of Cardiovascular and Pulmonary Sciences, Catholic University of the Sacred Heart, Rome, Italy; Center of Excellence in Cardiovascular Sciences, Isola Tiberina Hospital Gemelli Isola, Rome, Italy

**Keywords:** MINOCA, Stratified medicine, Angina, Intracoronary imaging, Cardiac magnetic resonance

## Abstract

**Background and Aims:**

Myocardial infarction with non-obstructive coronary arteries (MINOCA) is associated with a significant risk of mortality, rehospitalization, and angina burden. Despite its clinical impact, no randomized clinical trials have hitherto evaluated optimal management strategy for MINOCA. The PROMISE trial was designed to assess whether a stratified treatment improves clinical outcomes in patients with MINOCA as compared to standard care.

**Methods:**

PROMISE is a multicentre randomized trial. Patients with MINOCA were randomized 1:1 to either a stratified treatment based on a comprehensive diagnostic workup aimed at identifying the underlying aetiology, or to standard care. The primary endpoint was the between-group difference in the change in angina status at 12 months, assessed by the Seattle Angina Questionnaire summary score (SAQSS). The secondary endpoint was the incidence of major adverse cardiovascular events (MACE), defined as the composite of all-cause mortality, myocardial infarction, stroke, heart failure hospitalization and repeated coronary angiography. The trial was terminated early upon recommendation by the Data and Safety Monitoring Board due to clear benefits observed in the intervention group and potential harm in the control group.

**Results:**

Of 101 randomized patients, 92 were confirmed as MINOCA and included in the final analysis (mean age 62 ± 13 years, 48% women; stratified treatment *n* = 45; standard care *n* = 47). At 12-month follow-up, SAQSS was significantly higher in the stratified treatment than in standard care group, with a mean between-group difference of +9.38 in favour of the stratified treatment (95% confidence interval 6.81 to 11.95; *P* < .001). MACE occurred in 1 patient (2.2%) in the stratified treatment and in 4 patients (8.5%) in the standard care group, though the difference was not statistically significant (*P* = .18).

**Conclusions:**

In this first randomized trial of treatment strategies in MINOCA, a stratified treatment, based on comprehensive diagnostic assessment and aetiology-guided therapy, led to a significant improvement in angina-related health status. While the study findings provide the first evidence supporting individualized management in this heterogeneous and often under-recognized patient population, these results require confirmation in a larger prospective study with longer follow-up.

## Structured Graphical Abstract

**Graphical Abstract:**
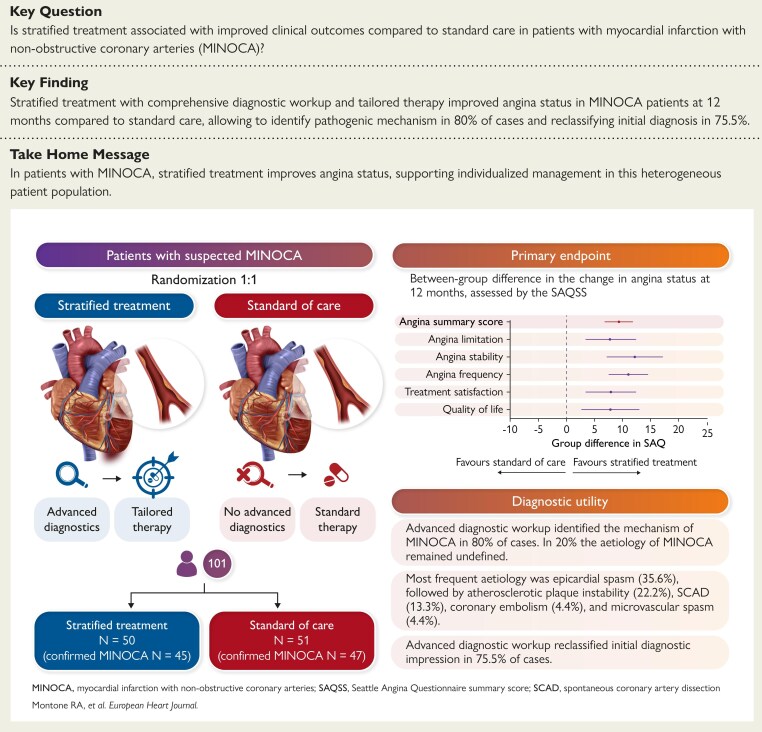
Study design with patient population, primary endpoint results, and assessment of diagnostic utility of the stratified treatment approach.

**See the editorial comment for this article ‘MINOCA: a call for randomized trials’, by C. Berry, https://doi.org/10.1093/eurheartj/ehaf1075.**

## Introduction

Myocardial infarction with non-obstructive coronary arteries (MINOCA) accounts for approximately 6%–8% of patients presenting with myocardial infarction (MI) who undergo coronary angiography.^1^ MINOCA is defined by clinical evidence of MI in the absence of obstructive coronary artery disease (i.e. no stenosis ≥50% in any major epicardial vessel) after exclusion of non-cardiac causes of MINOCA (i.e. pulmonary embolism, sepsis, severe anaemia, etc.), cardiac non-coronary causes (i.e. tachyarrhythmias, bradyarrhythmias, myocarditis etc) and Takotsubo syndrome as the ischaemic origin is still controversial.^2,3^ Initially described over 75 years ago, MINOCA is now recognized not as a single pathologic entity but as an umbrella term encompassing a broad spectrum of heterogeneous underlying mechanisms, such as atherosclerotic plaque instability (i.e. rupture or erosion), epicardial or microvascular spasm, spontaneous coronary artery dissection (SCAD), coronary thromboembolism.^4,5^ The pathophysiologic diversity of MINOCA presents a major clinical challenge, often requiring advanced diagnostic tools to determine the underlying aetiology.^6,7^ Importantly, MINOCA is not a benign clinical condition, as it is associated with considerable risk of recurrent ischaemic events, persistent angina, rehospitalization, and mortality.^8,9^ Yet, no prospective randomized clinical trial has been hitherto conducted to guide evidence-based management, and current treatment recommendations rely only on observational studies and expert consensus.^10^

To address this critical gap, we designed the ‘PROgnostic Value of Precision Medicine in Patients With Myocardial Infarction and Non-obStructive Coronary artEries’ (PROMISE) trial (ClinicalTrials.gov: NCT05122780). This investigator-initiated, randomized trial was conducted to evaluate whether a stratified therapeutic strategy guided by the knowledge of MINOCA aetiology, improves clinical outcomes in patients with MINOCA as compared to standard care.

## Methods

### Trial design and oversight

The PROMISE trial was a randomized, multicenter, prospective, open-label, superiority, phase IV trial comparing a ‘stratified treatment’ vs ‘standard of care’ approach in patients with MINOCA. The study design has been published previously,^11^ and the full protocol is available in the Supplementary data online, *Appendix*. The trial was funded by the Italian Ministry for Health and approved by the Italian Medicines Agency (AIFA) and the institutional review board at each participating center. All the patients provided written informed consent.

An independent, external Clinical Events Committee (CEC), blinded to treatment allocation, directly administered the Seattle Angina Questionnaire (SAQ) to all patients during follow-up to ensure unbiased data collection. Furthermore, CEC adjudicated all clinical outcomes included in the secondary endpoint. The authors had access to the trial data and vouched for the completeness and accuracy of the data and for the fidelity of the trial to the protocol. The initial draft of the manuscript was written by the first author.

### Patients

Eligible patients were adults hospitalized with a working diagnosis of MINOCA undergoing invasive coronary angiography. MINOCA was defined by the following criteria: (ⅰ) acute MI according to the Fourth Universal Definition of MI^2^; (ⅱ) non-obstructive coronary artery disease (i.e. no stenosis ≥50% in any major epicardial vessel). Exclusion criteria included: (ⅰ) contraindication to contrast-enhanced cardiac magnetic resonance imaging (CMRI) (e.g. glomerular filtration rate <30 mL/min or incompatible implantable devices); (ⅱ) end-stage renal and/or hepatic disease; (ⅲ) comorbidities with life expectancy <1 year; (ⅳ) presence of non-cardiac or cardiac non-coronary causes of MI; (ⅴ) Takotsubo syndrome; (ⅵ) MINOCA mechanism already evident at baseline coronary angiography (i.e. SCAD type 1). Full eligibility and exclusion criteria are provided in the Supplementary data online, *Appendix*.

Written informed consent was obtained prior to enrolment in the study and before any activity related to experimentation. In cases where the acute setting precluded immediate consent, verbal consent was initially obtained, followed by written consent as soon as feasible. Randomization was performed immediately after invasive coronary angiography, confirming the absence of obstructive coronary artery disease, using a secure, web-based randomization tool available 24/7 at any of the enrolling sites.

### Trial procedures, randomization and treatment

**Figure 1:**
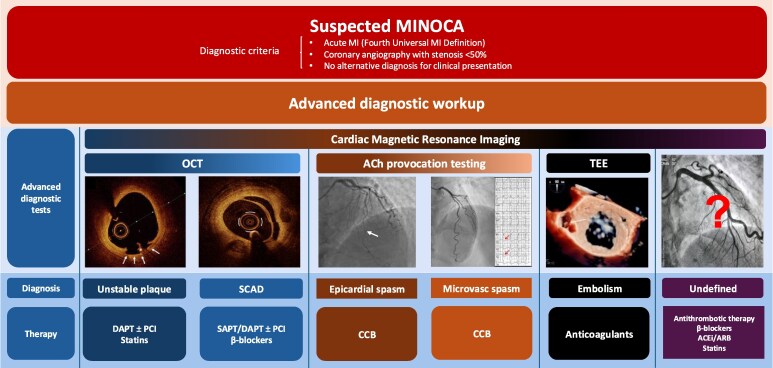
Stratified treatment in MINOCA patients. ACh, acetylcholine; CCB, calcium channel blockers; DAPT, dual antiplatelet therapy; OCT, optical coherence tomography; PCI, percutaneous coronary intervention; SAPT, single antiplatelet therapy; SCAD, spontaneous coronary artery dissection; TEE, transesophageal echocardiography

Patients were randomized 1:1 to either: (ⅰ) ‘stratified treatment’, consisting of a comprehensive diagnostic workup aimed at elucidating the pathophysiological mechanism of MINOCA and consequently treated with a tailored pharmacological approach; or (ⅱ) ‘standard of care’, consisting of the standard diagnostic algorithm and therapy for MI (*Figure 1*). Patients were enrolled in 4 large tertiary University Hospitals in Italy. Prior to randomization, the attending clinician was required to record the suspected aetiology of MINOCA based on initial clinical assessment.

In the ‘stratified treatment’ arm, patients underwent a comprehensive diagnostic workup (see Supplementary data online, *Figure S2*) aiming at identifying MINOCA aetiology consisting of: optical coherence tomography (OCT), if plaque instability was suspected based on coronary angiogram findings; intracoronary acetylcholine (ACh) provocation testing to assess the presence of coronary vasomotor disorders; transesophageal and/or contrast-enhanced echocardiography if an embolic aetiology was suspected based on the presence of risk factors for thromboembolism (e.g. atrial fibrillation, mechanical valves, thrombophilic disorders); CMRI. Aetiologies of MINOCA included atherosclerotic plaque instability (plaque rupture or erosion), SCAD, epicardial or microvascular spasm, coronary embolism. Afterwards, a targeted pharmacological treatment specific to the underlying cause was established (*Figure 1*). If the diagnostic workup still failed to identify a specific aetiology (‘undefined’ MINOCA), patients were managed with a comprehensive empirical regimen that included: antithrombotic therapy, tailored based on clinical judgment [single antiplatelet therapy (SAPT), dual antiplatelet therapy (DAPT), or oral anticoagulation if indicated]; beta-blockers and renin-angiotensin system inhibitors (angiotensin-converting enzyme inhibitors or angiotensin receptor blockers), for their cardioprotective and anti-ischemic properties (if tolerated); and statins, targeting potential underlying atherosclerotic disease or endothelial dysfunction.

Conversely, in the ‘standard of care’ arm, patients received a routine diagnostic workup without additional diagnostic tests (neither intracoronary imaging nor functional testing), and CMRI at the discretion of clinicians. Then, a standard medical treatment was established, consisting of SAPT or DAPT in all patients, along with other guidelines-directed prescriptions (*Figure 1*).

Patients were excluded from the study post-randomization if a specific non-MINOCA diagnosis was subsequently confirmed (e.g. myocarditis or Takotsubo syndrome).

### Outcomes and follow-up

The primary endpoint was the between-group difference in the change in angina status at 12 months, assessed by the Seattle Angina Questionnaire summary score (SAQSS). The secondary endpoint was the incidence of major adverse cardiovascular events (MACE) at 12 months, defined as the composite of all-cause mortality, non-fatal MI, stroke, rehospitalization for heart failure, and repeated coronary angiography.

### Statistical analysis

To detect a mean between-group difference of change in SAQSS of 9 points, we estimated that a sample size of 70 patients per group (140 patients in total) would provide 80% power at a two-sided significance level of 0.05, assuming a standard deviation (SD) of 19 units, consistent with prior literature.^12^ To account for potential dropouts or non-compliance, we initially extended the sample size to 180 patients. However, due to the slower-than-anticipated enrolment rate during the COVID-19 pandemic (2021–2022), and given that no patients were lost to follow-up (unlike what was initially anticipated), the study protocol was amended to reduce the sample size to 145 patients, which maintained the planned 80% statistical power to detect a 9-point between-group difference in SAQSS.^11^ At 3-year DSMB meeting (June 2024), an interim analysis was conducted after the enrolment of 92 confirmed MINOCA patients, and significance thresholds for the primary endpoint were evaluated using the O’Brien–Fleming approach (since this was the only interim look, the corresponding boundary was equivalent to the two-sided *α* = 0.05 with *k* = 2 and *Z* = 2.74). The DSMB recommended to stop the trial enrolment on the basis of: (ⅰ) clear benefit in the primary endpoint in the ‘stratified treatment’ arm (observed difference in SAQSS between groups was highly significant [*t* = 7.25, *P* < .0001], with the corresponding *Z*-score [6.42] exceeding the O’Brien–Fleming boundary [Z = 2.74], confirming the robustness of the result and a low risk of false-positive conclusion due to interim testing); (ⅱ) a numerically higher rate of MACE in the ‘standard of care’ arm; (ⅲ) alignment with the updated 2023 ESC Guidelines supporting systematic diagnostic evaluation in patients with suspected MINOCA (class I, level of evidence C).^10^

Continuous outcome measures assessed at baseline and 12 months were analyzed using a constrained longitudinal data analysis (cLDA) model, implemented as a linear mixed-effects regression including a random effect for patients and fixed effects for time (baseline and 12 months), randomized group, and their interaction. The baseline-adjusted intervention effect was estimated as the time-by-group interaction term from this model^13,14^ Categorical outcomes were compared between randomized groups using Chi-square or Fisher’s exact tests, as appropriate. Paired categorical comparisons of suspected vs confirmed aetiologies were assessed using the McNemar test for binary data. All analyses were two-sided, and a *P*-value ≤.05 was considered statistically significant. Continuous data are reported as mean ± SD or median with interquartile range, as appropriate. Statistical analyses were performed with the use of SPSS software, version 21 (IBM), and Stata software, version 17.0 (StataCorp). Analyses of secondary outcomes were not adjusted for multiplicity.

## Results

### Patients

Between 1 July 2021, and 30 June 2024, a total of 101 patients with a working diagnosis of MINOCA were enrolled and underwent randomization: 50 patients were assigned to the stratified treatment group and 51 to the standard of care group. Following, 9 patients were excluded due to the identification of an alternative non-MINOCA diagnosis during hospitalization (*n* = 4 Takotsubo syndrome, *n* = 5 myocarditis). Thus, 92 patients with a confirmed diagnosis of MINOCA were included in the final trial analysis: 45 in the stratified treatment group and 47 in the standard of care group. The study flow-chart is reported in Supplementary data online, *Figure S1*. Baseline demographic and clinical characteristics of the 92 patients are presented in *Table 1*, while therapy at discharge in *Table 2*. The mean age was 62 ± 13 years, and 48% were women. Cardiac MRI was performed in 77% of patients. The baseline SAQSS did not differ significantly between the two groups.

**Table 1:** Baseline demographic and clinical characteristics for the randomized population overall, and by randomized treatment group

Characteristics	Overall population (*n* = 92)	Stratified treatment (*n* = 45)	Standard of care (*n* = 47)
***Clinical characteristics***			
Age, years	61.8 ± 12.7	60.6 ± 13.1	62.8 ± 12.3
Female	44 (47.8)	18 (40.0)	26 (55.3)
Current smoker	33 (35.9)	21 (46.7)	12 (25.5)
Dyslipidaemia	37 (40.2)	17 (37.8)	20 (42.6)
Hypertension	50 (54.3)	21 (46.7)	29 (61.7)
Diabetes mellitus	17 (18.5)	8 (17.8)	9 (19.1)
COPD	10 (10.9)	4 (8.9)	6 (12.8)
IHD	2 (2.2)	0 (0.0)	2 (4.3)
CKD	2 (2.2)	1 (2.2)	1 (2.1)
Cerebrovascular disease	2 (2.2)	1 (2.2)	1 (2.1)
Clinical presentation			
STEMI	18 (19.6)	10 (22.2)	8 (17.0)
NSTEMI	74 (80.4)	35 (77.8)	39 (83.0)
Cardiac MRI performed	71 (77.2)	36 (80.0)	35 (74.5)
LVEF at admission, %	58 [55; 60]	58 [55; 60]	57 [55; 60]
***Laboratory data***			
Peak hs-cTnI (ng/mL)	1138.5 [138; 6994.7]	717.0 [140.5; 6361.0]	1867.0 [125; 7328.0]
Peak hs-CRP (ng/mL)	6.9 [2.2; 25.2]	8.0 [1.8; 27.3]	6.2 [2.2; 20.7]
Serum creatinine (mg/dL)	0.8 [0.7; 1.0]	0.8 [0.7; 0.9]	0.8 [0.7; 1.0]
Total cholesterol (mg/dL)	164 [135; 202]	164 [135; 187]	161.5 [131; 204]
LDL cholesterol (mg/dL)	96 [74.2; 126]	92.5 [78.2; 118]	99.5 [72; 134]

Values are median (InterQuartile Range), *n* (%), or mean ± Standard Deviation.

Abbreviations: BPCO, Chronic Obstructive Pulmonary Disease; CKD, Chronic Kidney Disease; hs-CRP, High-Sensitivity C-Reactive Protein; hs-cTnI, High Sensitivity Cardiac Troponin I; IHD, Ischemic Heart Disease; LDL, Low-Density Lipoprotein; LVEF, Left Ventricular Ejection Fraction; MRI, Magnetic Resonance Imaging; NSTEMI, Non-ST-Elevation Myocardial Infarction; STEMI, ST-Elevation Myocardial Infarction.

**Table 2:** Discharge therapy for the randomized population overall, and by randomized treatment group

Therapy	Overall population (*n* = 92)	Stratified treatment (*n* = 45)	Standard of care (*n* = 47)	*P* value
SAPT	31 (33.7)	16 (35.6)	15 (31.9)	.712
DAPT	29 (31.5)	15 (33.3)	14 (29.8)	.714
Beta-blockers	54 (58.7)	22 (48.9)	32 (68.1)	.062
CCB^a^	35 (38.0)	19 (42.2)	16 (34.0)	.419
Non-dihydropyridine	16 (17.1)	15 (33.3)	1 (2.1)	**<**.**001**
Dihydropyridine	21 (22.8)	6 (13.3)	15 (31.9)	.052
ARBs/ACEi	55 (59.8)	24 (53.3)	31 (66.0)	.217
Statins	75 (81.5)	38 (84.4)	37 (78.7)	.480
Nitrates	4 (4.3)	3 (6.7)	1 (2.1)	.356
Anticoagulant	18 (19.6)	7 (15.6)	11 (23.4)	.343

Values are *n* (%).

Abbreviations: ACEi, Angiotensin-Converting Enzyme Inhibitors; ARBs, Angiotensin II Receptor Blockers; CCB, Calcium Channel Blockers; DAPT, Dual Antiplatelet Therapy; SAPT, Single Antiplatelet Therapy.

^a^Details on CCB prescription. In the Standard of care group, the use of CCB predated MINOCA admission in 15 patients and was maintained at discharge; in 15 patients taking CCB and allocated to standard of care the indication for CCB use was hypertension (dihydropyridine) and in 1 patient was supraventricular arrhythmias (non-dihydropyridine).

In the Stratified treatment group, the indication for CCB was ‘coronary spasm’ in 16 patients (15 non-dihydropyridine and 1 dihydropyridine), while in 3 patients the indication was hypertension. 2 patients with coronary spasm were discharged with a combination of non-dihydropyridine and dihydropyridine.

### Primary endpoint

All enrolled patients completed the primary endpoint assessment (SAQSS).

**Figure 2:**
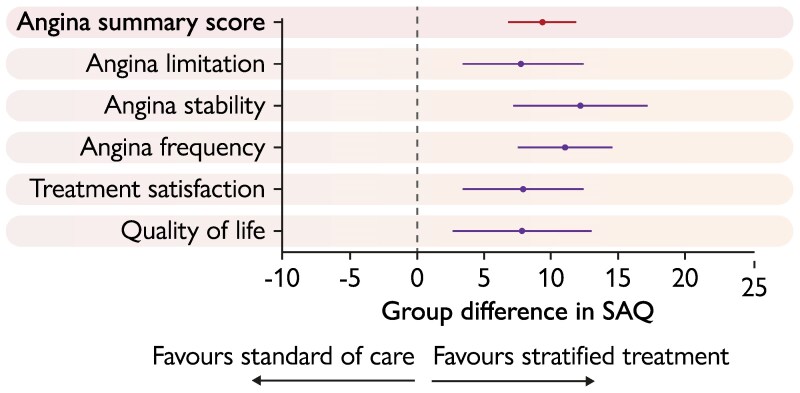
Primary endpoint

SAQSS at 12 months was significantly higher in the stratified treatment group than in the standard of care group (82.7 ± 7.3 vs 74.7 ± 10.3, *P* < .001). The ‘stratified treatment’ approach resulted in an improvement of 9.38 points [95% confidence interval (CI): 6.81 to 11.95; *P* < .001] greater than the standard of care approach, and this difference was consistent across all five domains of the SAQ, including physical limitation, angina stability, angina frequency, treatment satisfaction, and quality of life (*Table 3* and *Figure 2*). Angina-free patients at 12 months were 64 (71.1%) in the overall population, with a borderline significance for a higher number of patients angina-free in the stratified treatment group compared with the standard of care group of [36 (80.0%) vs 28 (62.2%), *P* = .06, respectively]. Therapies at 12 months in the two study groups and according to aetiology in the stratified treatment group are reported in Supplementary data online, *Tables S1* and *S2*.

**Table 3:** Primary outcome at 12 months

Primary Outcome—Seattle Angina Questionnaire	Stratified treatment (*n* = 45)		Standard of care (*n* = 47)		Intervention Effect		
	12 months	Change From Baseline	12 months	Change From Baseline	Estimate	95% CI	*P* Value
Angina summary score	82.7 ± 7.3	12.3 ± 5.2	74.7 ± 10.3	2.9 ± 9.5	9.38	6.81 to 11.95	**<**.**001**
Angina limitation	91.7 ± 13.6	13.1 ± 11.5	84.3 ± 17.5	8.7 ± 20.1	7.84	3.22 to 12.46	.**001**
Angina stability	65.0 ± 21.6	11.1 ± 18.9	56.4 ± 15.2	4.2 ± 24.5	12.17	7.11 to 17.23	**<**.**001**
Angina frequency	96.4 ± 7.1	13.8 ± 10.9	91.5 ± 14.0	6.4 ± 19.4	11.01	7.39 to 14.63	**<**.**001**
Treatment Satisfaction	82.3 ± 13.9	13.8 ± 13.2	74.7 ± 16.1	8.9 ± 22.2	7.96	3.51 to 12.40	**<**.**001**
Quality of life	78.0 ± 17.6	9.8 ± 17.8	66.7 ± 18.3	5.3 ± 22.9	7.87	2.68 to 13.06	.**003**

### Secondary endpoint

At 12 months, MACE occurred in five patients, with no significant difference between the two study groups [4 (8.5%) in the standard of care group vs 1 (2.2%) in the stratified treatment group; *P* = .186]. In particular, standard of care patients experienced 2 (4.3%) deaths, 1 (2.1%) stroke and 1 (2.1%) rehospitalization for heart failure, while 1 (2.2%) patient allocated to stratified treatment experienced a non-fatal MI (*Table 4*). Of note, 2 deaths occurring in the standard of care group were cardiac (1 patient died for a cardiac arrest and 1 patient during an episode of acute heart failure, both in hospital, not during index MINOCA admission but during follow-up).

**Table 4:** Secondary Clinical Outcomes at 12 Months

Clinical events	Overall population (*n* = 92)	Stratified treatment (*n* = 45)	Standard of care (*n* = 47)	*P* value
MACE	5 (5.4)	1 (2.2)	4 (8.5)	.186
All-cause Mortality	2 (2.2)	0 (0.0)	2 (4.3)	.160
Non-fatal MI	1 (1.1)	1 (2.2)	0 (0.0)	.317
Stroke	1 (1.1)	0 (0.0)	1 (2.1)	.323
Rehospitalization For Heart Failure	1 (1.1)	0 (0.0)	1 (2.1)	.328
Repeated Coronary Angiography	0 (0.0)	0 (0.0)	0 (0.0)	-

Values are *n* (%).

Abbreviations: MACE, Major Adverse Cardiovascular Events; MI, Myocardial Infarction.

### Safety and adverse events

No serious adverse events secondary to the advanced diagnostic workup occurred in the stratified treatment group. Furthermore, no serious adverse events linked to medical therapy were registered in both study groups.

### Diagnostic utility

**Figure 3:**
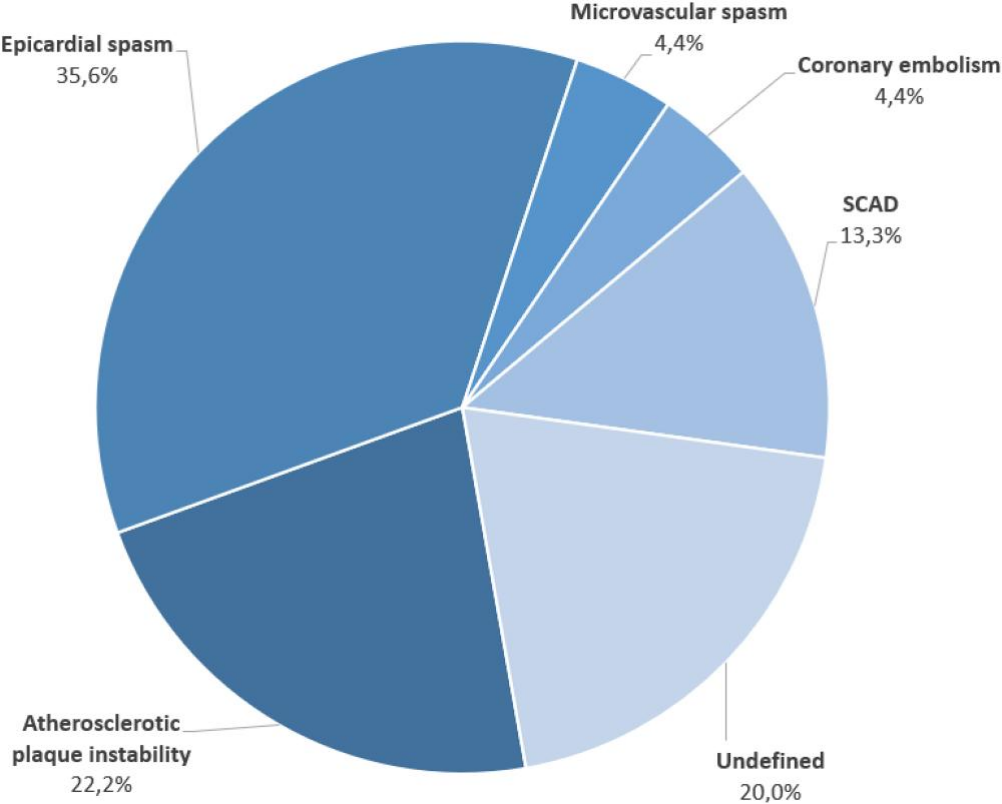
Aetiologies of MINOCA in the stratified treatment group

In the stratified treatment group, the diagnostic workup successfully identified the underlying aetiology of MINOCA in 36 out of 45 patients (80%) (*Figure 3*). The most frequent aetiology was epicardial spasm, detected in 16 patients (35.6%), followed by atherosclerotic plaque instability in 10 patients (22.2%), SCAD in 6 patients (13.3%), coronary embolism in 2 patients (4.4%), and microvascular spasm in 2 patients (4.4%). In 9 patients (20%), the aetiology of MINOCA remained undefined. Of note, all patients with an undefined aetiology underwent CMRI. Discharge therapies according to specific aetiologies are reported in *Table 5*.

**Table 5:** Discharge therapies according to MINOCA aetiology in the stratified treatment group

Therapy	Atherosclerotic Plaque Instability (*n* = 10)	Epicardial spasm (*n* = 16)	Microvascular spasm (*n* = 2)	Coronary embolism (*n* = 2)	SCAD (*n* = 6)	Undefined (*n* = 9)
SAPT	1 (10.0)	9 (56.3)	0 (0.0)	0 (0.0)	2 (33.3)	4 (44.4)
DAPT	9 (90.0)	0 (0.0)	0 (0.0)	0 (0.0)	4 (66.7)	2 (22.2)
Beta-blockers	7 (70.0)	0 (0.0)	1 (50.0)	1 (50.0)	5 (83.3)	8 (88.9)
CCB	1 (10.0)	16 (100.0)	0 (0.0)	0 (0.0)	1 (16.7)	0 (0.0)
ARBs/ACEi	6 (60.0)	6 (37.5)	1 (50.0)	1 (50.0)	4 (66.7)	6 (66.7)
Statins	9 (90.0)	13 (81.3)	2 (100.0)	2 (100.0)	4 (66.7)	8 (88.9)
Nitrates	0 (0.0)	2 (12.5)	0 (0.0)	0 (0.0)	0 (0.0)	1 (11.1)
Anticoagulant	2 (20.0)	0 (0.0)	0 (0.0)	2 (100.0)	0 (0.0)	3 (33.3)
PCI^a^	8 (80.0)	0 (0.0)	0 (0.0)	0 (0.0)	3 (50.0)	0 (0.0)

Values are *n* (%).

Abbreviations: ACEi, Angiotensin-Converting Enzyme Inhibitors; ARBs, Angiotensin II Receptor Blockers; CCB, Calcium Channel Blockers; DAPT, Dual Antiplatelet Therapy; PCI, Percutaneous Coronary Intervention; SAPT, Single Antiplatelet Therapy.

^a^Decision to perform PCI was left per protocol to operator’s choice and was performed for recurrence or persistence of angina/ischemia in 3 patients with SCAD and to passivate a high-risk plaque in 8 patients with atherosclerotic plaque instability.

When comparing the pre-advanced diagnostic workup suspected aetiology with the final diagnosis established after the diagnostic workup, the initial diagnostic impression was reclassified in 34 patients (75.5%). The distribution of reclassified diagnoses by specific aetiologies is reported in *Table 6*.

**Table 6:** Diagnostic Utility of a Stratified Medicine Approach in Reclassifying MINOCA Aetiology in the ‘Stratified treatment’ group

MINOCA aetiology	Suspected Diagnosis (pre-advanced diagnostic work-up)	Confirmed Diagnosis (post-advanced diagnostic work-up)	Reclassified^a^ Diagnosis	*P* value
Atherosclerotic Plaque Instability	27 (60.0)	10 (22.2)	17 (37.8)	**<**.**001**
Epicardial spasm	6 (13.3)	16 (35.6)	10 (22.2)	.**006**
Microvascular spasm	0 (0.0)	2 (4.4)	2 (4.4)	.500
Coronary embolism	2 (4.4)	2 (4.4)	0 (0.0)	1.000
SCAD	4 (8.9)	6 (13.3)	2 (4.4)	.500
Undefined	6 (13.3)	9 (20.0)	3 (6.7)	.289

Values are *n* (%).

^a^Defined as the number and percentage of patients with a different confirmed vs suspected aetiology.

Abbreviations: SCAD, Spontaneous Coronary Artery Dissection.

In a sensitivity analysis performed for the primary endpoint after excluding patients with an undefined MINOCA, the stratified treatment still yielded a 9.61 points improvement in SAQSS compared with standard of care (95% CI: 6.84 to 13.38; *P* < .001).

## Discussion

The main findings of the PROMISE trial can be summarized as follows: (ⅰ) in patients with MINOCA, a stratified treatment, comprising a structured diagnostic workup followed by aetiology-specific treatment, led to an improvement in angina-related health status at 12 months compared with standard care, and this benefit was consistent across all five domains of the SAQ; (ⅱ) the incidence of MACE did not differ between the stratified treatment group and the standard of care group, even though the trial was not powered to detect differences in clinical events; (ⅲ) the stratified treatment proved feasible and safe, with no adverse events related to the diagnostic tests or medical therapy; (ⅳ) importantly, the stratified treatment resulted in reclassification of the initial suspected diagnosis in 75.5% of cases, highlighting its diagnostic value (*Structured Graphical Abstract*).

To our knowledge, the PROMISE trial represents the first prospective, multicentre, randomized clinical trial specifically conducted in patients with MINOCA. The study demonstrates that a stratified treatment, consisting of structured diagnostic workup and aetiology-guided therapy, leads to clinically meaningful improvements in angina-related health status. Although baseline SAQ summary scores were relatively high, the mean improvement of approximately 10 points exceeds the established minimal clinically important difference for the SAQ (≥5 points) and therefore remains clinically relevant even within this high-range baseline.^15^ Persistent angina is a clinically significant and overlooked issue in the MINOCA population, with both high prevalence and prognostic implications. Previous studies demonstrated that approximately 25% of patients continue to experience angina within the first year after the index event, with reduced quality of life and increased healthcare utilization.^16^ The socioeconomic impact is also considerable, as these patients experience rates of disability pension and premature workforce exit comparable to those with obstructive coronary artery disease.^17^ This issue is particularly relevant given the younger age profile of many individuals with MINOCA, making angina not only a clinical challenge but also a public health and economic concern.

The CorMicA trial^12^ provided the first evidence for a benefit deriving from a stratified medicine approach in stable patients with ischemia and non-obstructive coronary arteries (INOCA) and informed subsequent expert consensus paper and clinical guidelines.^18,19^ Similarly, our findings provide the first randomized evidence supporting the rationale for individualized, mechanism-driven management in MINOCA, directly addressing a critical gap in the existing evidence base. Indeed, recommendations of current acute coronary syndromes guidelines have largely relied on observational data and expert consensus, as reflected by the Class I, Level of Evidence C recommendation for systematic diagnostic algorithms and the Class I, Level B for aetiology-specific treatment.^10^ The large SWEDEHEART registry reported associations between statin or renin-angiotensin-aldosterone system inhibitor use and improved long-term outcomes in MINOCA patients, whereas DAPT, a cornerstone of MI with obstructive coronary artery disease, did not offer any benefit. Of note, this study lacked information on the underlying pathophysiology and was unable to control for unmeasured confounding, limiting its ability to inform aetiology-specific treatment strategies.^20^ By providing, for the first time, high-quality randomized evidence supporting a structured diagnostic and therapeutic strategy, the PROMISE trial may inform future updates in guidelines strength and clinical adoption.

The benefit of the stratified treatment is likely attributable to its ability to define the underlying mechanism of MINOCA and deliver appropriate treatment. In the stratified treatment group, the specific cause of MINOCA was identified in 80% of patients, and the initial clinical suspicion was reclassified in over 75% of cases. This high diagnostic yield underscores the limitations of syndromic labelling and the risks of empirical, non-specific treatment. Indeed, patients in the standard care arm were managed according to conventional post-MI protocols, most receiving uniform regimens including antiplatelet therapy, statins, and beta-blockers, irrespective of the underlying pathophysiology. While such therapy may be appropriate for some aetiologies (e.g. plaque instability), it is suboptimal or even potentially harmful in others (e.g. beta-blockers may increase the risk of recurrent events in vasospastic angina; SAPT or DAPT are inadequate in case of coronary embolism).^6^ Furthermore, the significantly greater improvement in angina-related health status observed in the stratified treatment group likely reflects not only more effective and targeted treatment, but also the reduced diagnostic uncertainty for both patients and clinicians. Of importance, PROMISE trial also confirmed the feasibility and safety of implementing advanced diagnostic techniques, including intracoronary imaging and vasomotor testing, into routine clinical workflows. Indeed, no adverse events related to these invasive procedures or to therapy were observed, reinforcing existing evidence supporting their safety, even when applied in the acute setting.

Moreover, the PROMISE trial provides the first data on the prevalence of different underlying mechanisms in MINOCA, employing what is arguably the most comprehensive and standardized diagnostic workup to date. Epicardial spasm was the most common aetiology, being identified in approximately one-third of patients (35.6%), followed by atherosclerotic plaque instability (22.2%), SCAD (13.3%), and less frequently, coronary embolism and microvascular spasm (each <5%). These findings differ from those of previous observational studies, which have generally reported a higher prevalence of plaque disruption and a lower prevalence of vasospastic mechanisms, differences that likely derive from variability in diagnostic protocols and completeness of evaluation.^21–23^ In this regard, a recent meta-analysis by Fedele *et al*.^24^ reported that OCT and vasoreactivity testing can frequently identify an underlying cause in MINOCA, with rates of culprit plaque and positive ACh testing reported at 62% and 49%, respectively. However, the analysis was limited by high heterogeneity in definitions, study populations, and testing protocols, and by the lack of systematic multimodal assessment, which may have led to an underestimation of overlapping or multifactorial mechanisms. Similarly, Zeng *et al*.^23^ reported that atherosclerotic causes identified by OCT were present in 52% of MINOCA patients and associated with a worse 1-year prognosis.^23^ However, the absence of adjunctive tests such as CMRI or provocative testing led to 38.9% of cases being unclassified. Additionally, the primary composite endpoint was heavily influenced by soft events such as rehospitalization and target lesion revascularization, potentially biasing results toward atherosclerotic findings. The HARP study adopted a more integrated diagnostic strategy, combining OCT and CMRI in women with MINOCA and achieving an 84.5% diagnostic yield.^25^ Nonetheless, it was limited by its observational nature, inclusion of women only, and lack of clinical outcome assessment. Furthermore, in the HARP study the authors considered as culprit lesions also layered plaque and intraplaque cavity, whose pathogenic role in acute coronary syndromes is arguable, along with including conditions such as myocarditis and Takotsubo syndrome as potential causes of MINOCA, which are now considered exclusionary diagnoses based on contemporary definitions and were systematically excluded from the PROMISE.

Notably, despite the use of intracoronary imaging, vasomotor testing, and multimodal imaging, a definitive mechanism remained unidentified in 20% of patients. This residual diagnostic uncertainty has several important implications. First, it underscores the biological heterogeneity of MINOCA and reinforces the notion that MINOCA is not a single disease entity but a syndrome with diverse and sometimes elusive pathophysiologic processes. Second, it reflects real-world challenges, as not all diagnostic tests may be feasible or interpretable in every patient, whether due to clinical instability, anatomical limitations, or suboptimal image quality. Third, although CMRI was performed in approximately 80% of patients in PROMISE, this still leaves a proportion without the benefit of myocardial tissue characterization, further supporting recommendations for routine CMRI in all MINOCA cases when feasible. Finally, the fact that one in five patients remained without a definitive diagnosis even after a comprehensive evaluation underscores the urgent need for continued innovation in diagnostic strategies, possibly including emerging modalities such as hybrid imaging and molecular or biomarker profiling.

The early termination of the trial and the final sample size warrant consideration. Enrolment was slower than anticipated, partly due to the impact of the COVID-19 pandemic during 2021–2022. Although the protocol was amended to reduce the sample size to 145 patients without compromising statistical power, the trial was ultimately stopped following DSMB recommendation. This decision was based on the clear superiority of the precision medicine arm for the primary endpoint, a numerically higher rate of MACE in the standard of care arm, and evolving guideline supporting structured diagnostic evaluation in MINOCA. Notably, despite the reduced sample size, all enrolled patients completed 12-month follow-up, and the consistency of benefit across all domains of the SAQ, along with the diagnostic performance and safety data, strengthens the internal validity and external applicability of the findings. Of note, it should be considered that the PROMISE represents the first trial performed in MINOCA and its sample size was calculated based on the CorMiCa trial,^12^ enrolling stable patients with INOCA rather than MINOCA. It is conceivable that the more aggressive pathophysiologic mechanisms underlying the occurrence of MI in MINOCA patients may explain the significant results even with a smaller sample size. Furthermore, in the CorMicA trial baseline SAQSS was lower than in our trial. These differences between MINOCA and INOCA patients warrant further investigations in future studies.

### Limitations

This trial has several limitations that should be acknowledged. First, the sample size was relatively modest and not powered to detect differences in MACE or other secondary endpoints. Therefore, definitive conclusions regarding clinical event reduction cannot be drawn from this trial alone. Second, the open-label design may have introduced performance bias, particularly regarding patient-reported outcomes, considering also the reduced diagnostic uncertainty in the stratified treatment group for both clinicians and patients. However, the risk of detection bias was mitigated using a blinded CEC for endpoint adjudication, and the primary outcome (e.g. SAQSS) is a validated instrument with high reliability in assessing health status. Third, the study was conducted in four tertiary university hospitals in Italy, all with high levels of expertise in invasive coronary diagnostics and cardiac imaging. This may limit the generalizability of the findings to centres without access to advanced imaging modalities or specialized personnel. Broader implementation in more diverse healthcare systems will require consideration of resource availability, training, and cost-effectiveness. Fourth, the follow-up period was limited to 12 months. While this timeframe was adequate to assess angina-related health status, it may be insufficient to fully capture the long-term impact of aetiology-specific therapy on hard clinical outcomes such as recurrent MI, heart failure, or mortality. Longer follow-up would help clarify the durability of benefit and its effect on healthcare utilization. Another point concerns the apparent lack of difference in the rates of discharge calcium channel blocker (CCB) therapy between the two study groups. However, patients in the stratified treatment group were more frequently discharged on non-dihydropyridine CCBs and less frequently on beta-blockers, reflecting the management of newly diagnosed vasospasm. In contrast, patients in the standard of care group were mainly discharged on dihydropyridine CCBs prescribed for concomitant hypertension. Furthermore, percutaneous coronary intervention (PCI) was performed in 11 patients within the stratified treatment group. The decision to perform PCI was made at the discretion of the treating physician. Importantly, the results of this trial should not be interpreted as endorsing the routine use of PCI in patients with MINOCA secondary to SCAD or plaque instability.

Finally, the exclusion of patients in whom MINOCA was not confirmed during diagnostic workup, while methodologically appropriate, may introduce a degree of selection bias and limit applicability to patients with more ambiguous presentations in real-world settings.

## Conclusions

In the first randomized trial in MINOCA, a stratified treatment, based on comprehensive diagnostic assessment and aetiology-guided therapy, led to significant improvement in angina-related health status. These findings provide the first randomized evidence supporting individualized management in this heterogeneous and often under-recognized patient population. However, due to the small sample size and the open-label design, our results warrant further validation in future studies. Moreover, further research is warranted to assess the impact of this approach on hard clinical outcomes in larger cohorts and diverse healthcare settings.

## Supplementary Material

ehaf917_Supplementary_Data

## Declarations

### Data Availability

The data underlying this article will be shared on reasonable request to the corresponding author.

### Pre-registered Clinical Trial Number

The pre-registered clinical trial number is NCT05122780.
