# Supplementary material for: Stratified treatment of myocardial infarction with non-obstructive coronary arteries: the PROMISE trial
Source: Eur Heart J. 2025 Oct 28;47(12):1456–66. doi: 10.1093/eurheartj/ehaf917 (PMC13008472; doi:10.1093/eurheartj/ehaf917)
Supplement: ehaf917_Supplementary_Data [file ehaf917_supplementary_data.pdf]

Supplementary appendix

- **Study organization**
- **Committees and organization**
- **Manuscript responsibility**
- **Funding**
- **Inclusion and exclusion criteria**
- **Endpoint definitions**
- **Figure S1. Enrolment flow-chart**
- **Figure S2. Advanced diagnostic work-up sequence**
- **Table S1. Therapy at 12-month follow up by randomized treatment group**
- **Table S2. Therapy at 12-month follow up according to aetiology in the stratified treatment group**
- **Table S3. Advanced diagnostic work-up for each patient enrolled in the stratified treatment group**

STUDY ORGANIZATION

Site investigators

- **Fondazione Policlinico Universitario A. Gemelli IRCCS (Rome, Italy)**

  PI: Rocco A. Montone

  Investigators: Giulia La Vecchia, Alice Bonanni, Andrea Caffè, Vincenzo Scarica, Antonio Maria Leone, Tommaso Sanna, Giovanna Liuzzo, Carlo Trani, Francesco Burzotta, Filippo Crea.

- **Centro Cardiologico Monzino (Milan, Italy)**

  PI: Nicola Cosentino

  Investigators: Marta Resta, Claudia Lucci, Giancarlo Marenzi, Federico De Marco.

- **IRCCS Policlinico San Donato (San Donato Milanese, Milan, Italy)**

  PI: Riccardo Gorla

  Investigators: Luca Testa, Francesco Bedogni.

- **Azienda Ospedaliero Universitaria di Ferrara (Ferrara, Italy)**

  PI: Simone Biscaglia

  Investigators: Gianluca Campo, Andrea Erriquez.

COMMITTES AND ORGANIZATION

Data Safety Monitoring Board

Chair: Prof. Fabio Mangiacapra (University Campus Biomedico, Rome, Italy)

Member: Dr. Giulia Magnani (University of Parma, Italy)

Clinical Event Committee

Chair: Prof. Giampaolo Niccoli (University of Parma, Italy)

Member: Dr. Filippo Gurgoglione (University of Parma, Italy)

Executive Committee Members

Study Principal Investigator: Dr. Rocco A. Montone

Study Chair: Prof. Filippo Crea

Members: Simone Biscaglia, Riccardo Gorla, Nicola Cosentino.

Executive statistician

Riccardo Rinaldi (Cardiovascular Clinic Institute, Hospital Clínic, University of Barcelona, Spain).

MANUSCRIPT RESPONSIBILITY

Rocco A. Montone wrote the first draft of the manuscript, which was critically revised and checked for consistency by the Executive Committee members and the members of the Statistical Committee. All remaining authors critically revised the manuscript. Rocco A. Montone submitted the manuscript for publication on behalf of the authors.

FUNDING

The study was supported by a Research Grant awarded to the Principal Investigator Dr. Rocco A. Montone from the Italian Ministry of Health. Grant "Ricerca Finalizzata 2019" for Young Investigators. Grant number: GR-2019-12370197.

The funding institution was not involved with the study processes, including site selection and management, and data collection and analysis.

INCLUSION AND EXCLUSION CRITERIA

Inclusion criteria:

– Ability to give informed consent to the study

– Age ≥18 years

– MINOCA diagnosis, defined as:

  – Acute MI (based on the Fourth Universal Definition of Myocardial Infarction Criteria)

  – Evidence of non-obstructive coronary artery disease on CAG (i.e., no coronary artery stenosis >50%)

  – No specific alternate diagnosis for the clinical presentation.

Exclusion criteria:

- Age <18 years
- Pregnant and breast-feeding women or patients considering becoming pregnant during the study period.
- Alternate diagnosis for the clinical presentation.
- Contraindication to contrast-enhanced CMR (e.g., severe renal dysfunction [glomerular filtration rate <30 mL/min]) or non-CMR-compatible pacemaker/defibrillator.
- Contraindication to drugs administered: e.g., a history of hypersensitivity to drugs administered or its excipients, significant renal and/or hepatic disease.
- Patients with comorbidities having an expected survival <1 year will be excluded
- Inability or limited capacity to give informed consent to the study
- MINOCA mechanism already evident at baseline coronary angiography (i.e. SCAD type 1)

- presence of non-cardiac or cardiac non-coronary causes of MINOCA (anemia, hypertensive crisis, sepsis, pulmonary embolism, tachyarrhythmias, bradyarrhythmias, cardiac trauma, drug toxicity, etc.)
- Takotsubo syndrome
- myocarditis.

ENDPOINT DEFINITIONS

Primary endpoint

The primary endpoint was the between-group difference in the change in angina status at 12 months, assessed by the Seattle Angina Questionnaire summary score (SAQSS).

Secondary endpoint

The secondary endpoint was the incidence of major adverse cardiovascular events (MACE), defined as the composite of all-cause mortality, MI, stroke, heart failure hospitalization and repeated coronary angiography.

Myocardial infarction

The myocardial infarction outcome was defined based on the Fourth Universal Definition of myocardial infarction.

• Myocardial infarction type 1

Detection of a rise and/or fall of cTn (cardiac troponin) values with at least one value above the 99th percentile upper reference limit (URL) and with at least one of the following: o Symptoms of acute myocardial ischemia; o New ischemic ECG changes; o Development of pathological Q waves; o Imaging evidence of new loss of viable myocardium or new regional wall motion abnormality in a pattern consistent with an ischemic aetiology; o Identification of a coronary thrombus by angiography including intracoronary imaging or by autopsy.

• Myocardial infarction type 2

Detection of a rise and/or fall of cTn values with at least one value above the 99th percentile URL, and evidence of an imbalance between myocardial oxygen supply and demand unrelated

to acute coronary atherothrombosis, requiring at least one of the following: o Symptoms of acute myocardial ischemia; o New ischemic ECG changes; o Development of pathological Q waves; 12 o Imaging evidence of new loss of viable myocardium or new regional wall motion abnormality in a pattern consistent with an ischemic aetiology;

• Myocardial infarction type 3

Patients who experience cardiac death, with symptoms suggestive of myocardial ischemia accompanied by presumed new ischemic ECG changes or ventricular fibrillation but die before blood samples for biomarkers can be obtained, or before increases in cardiac biomarkers can be identified, or MI is detected by autopsy examination.

• Myocardial infarction type 4a

Coronary procedure-related MI ≤ 48 h after the index procedure is arbitrarily defined by an elevation of cTn values > 5 times the 99th percentile URL in patients with normal baseline values. Patients with elevated pre-procedure levels of cTn, in whom the pre-procedure cTn levels are stability (≤ 20% variation) or falling, must meet the criteria for a > 5-fold increase and manifest a change from the baseline value of > 20%. In addition, with at least one of the following: o New ischemic ECG changes; o Development of new pathological Q waves; o Imaging evidence of loss of viable myocardium that is presumed to be new and in a pattern consistent with an ischemic aetiology; o Angiographic findings consistent with a procedural flow-limiting complication such as coronary dissection, occlusion of a major epicardial artery or graft, side branch occlusion/thrombus, disruption of collateral flow, or distal embolization. o Isolated development of new pathological Q waves if cTn values are elevated and rising but less than pre-specified thresholds for PCI and CABG. o Post-mortem demonstration of a procedure-related thrombus.

• Myocardial infarction type 4b (stent thrombosis)

A subcategory of PCI-related MI is stent/scaffold thrombosis, as documented by angiography or autopsy using the same criteria utilized for type 1 MI. The time of occurrence of stent/scaffold 13 thrombosis relative to PCI should be indicated, and the following temporal categories are suggested: acute, 0–24 h; subacute, > 24 h to 30 days; late, > 30 days to 1 year; and very late > 1 year after stent/scaffold implantation.

• Myocardial infarction type 4c (restenosis)

Occasionally MI occurs and—at angiography, in-stent restenosis, or restenosis following balloon angioplasty in the infarct territory—is the only angiographic explanation since no other culprit lesion or thrombus can be identified. This PCI-related MI type is designated as type 4c MI, defined as focal or diffuse restenosis, or a complex lesion associated with a rise and/or fall of cTn values above the 99th percentile URL applying, the same criteria utilized for type 1 MI.

• Myocardial infarction type 5

CABG-related MI is arbitrarily defined as elevation of cTn values > 10 times the 99th percentile URL in patients with normal baseline cTn values. Patients with elevated pre-procedure levels of cTn, in whom the pre-procedure cTn levels are stability (≤ 20% variation) or falling, must meet the criteria for a > 10-fold increase and manifest a change from the baseline value of > 20%. In addition, one of the following elements is required: o Development of new pathological Q waves; Isolated development of new pathological Q waves meets the type 5 MI criteria if cTn values are elevated and rising but < 10 times the 99th percentile URL; o Angiographic documented new graft occlusion or new native coronary artery occlusion; o Imaging evidence of new loss of viable myocardium or new regional wall motion abnormality in a pattern consistent with an ischemic aetiology.

Stroke

Defined as the presence of a new focal neurologic deficit thought to be vascular in origin, with signs or symptoms lasting more than 24 hours. It is strongly recommended (but not required) that an imaging procedure such as CT scan or MRI be performed. Stroke will be further classified as ischemic, haemorrhagic, or type uncertain.

Hospitalization for heart failure

Hospitalization lasting more than 1 day due to the presence of signs and symptoms of heart failure.

Repeated coronary angiography

Coronary angiography due to angina and/or evidence of rest or inducible myocardial ischemia.

FIGURE S1. Enrolment flow-chart.

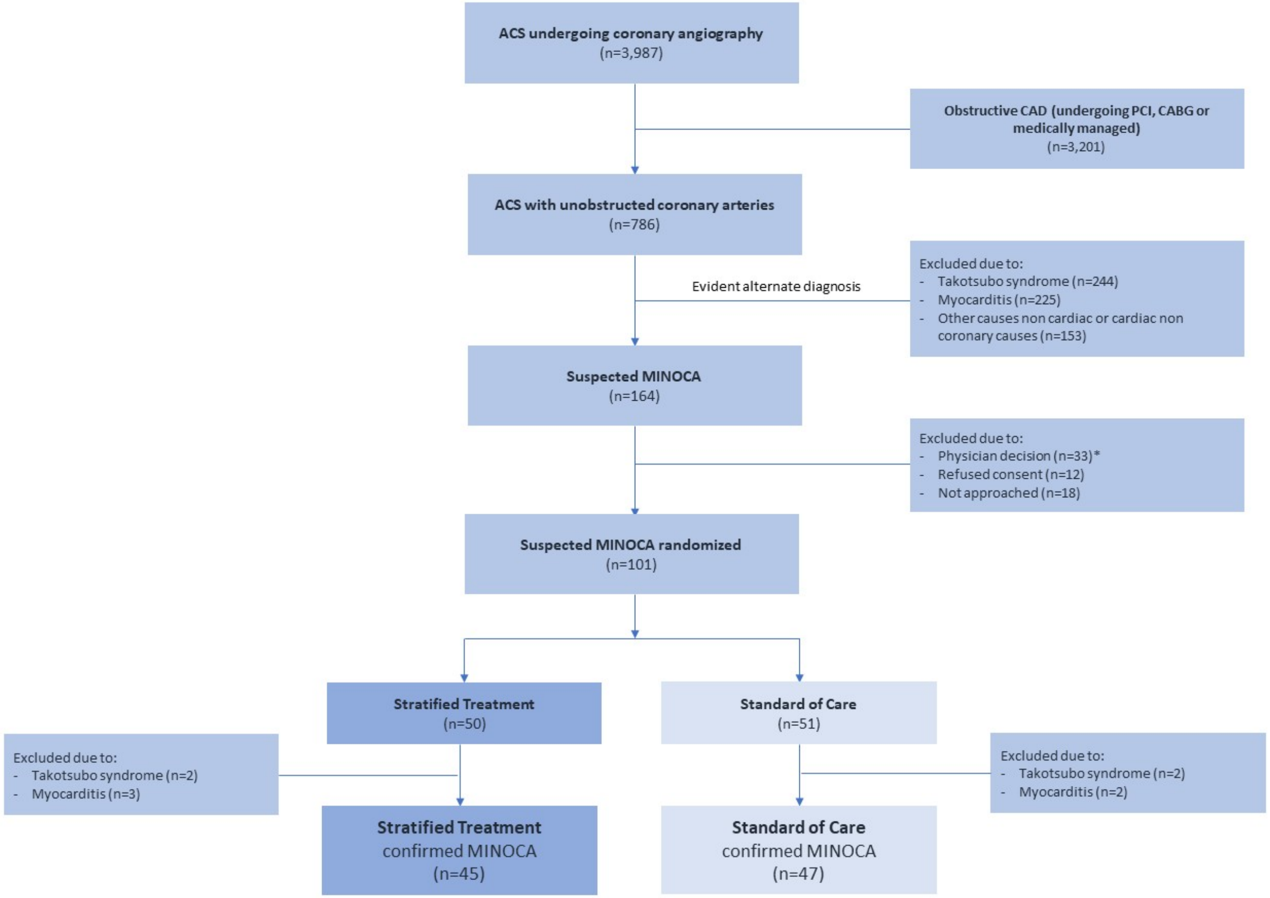

Legend: *patients excluded to physician decision include: 6 patients with SCAD type 1 already evident at baseline coronary angiography and confirmed by subsequent follow up coronary computed tomography at follow up; 4 patients with coronary anatomy judged not suitable for intracoronary imaging (extreme vessel tortuosity); 8 patients with advanced renal insufficiency; 4 patients with cancer history and expected survival < 1-year; 8 patients with haemodynamic instability; 3 patients with arrhythmic instability.

FIGURE S2. Advanced diagnostic work-up sequence.

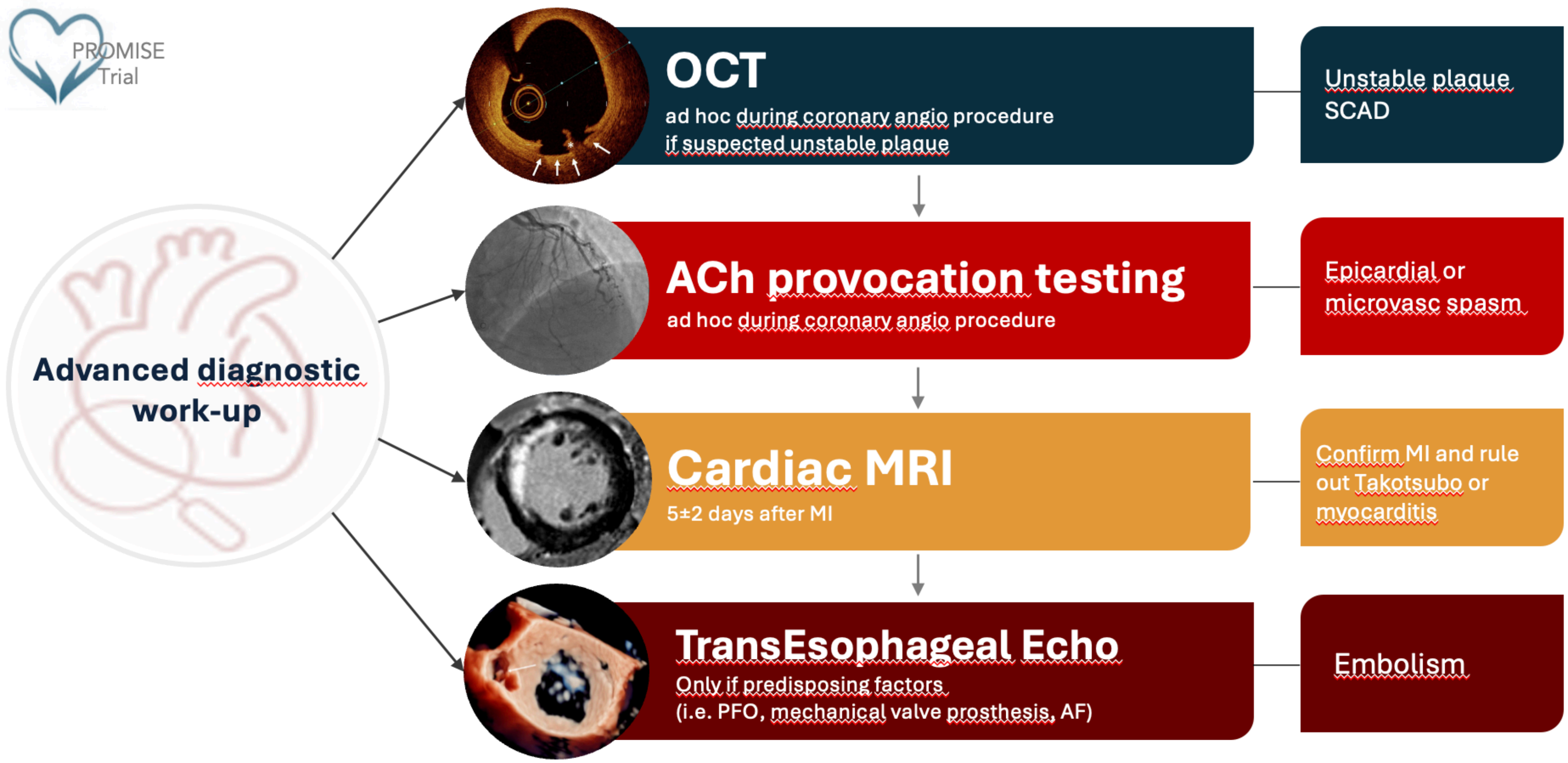

Legend: ACh: acetylcholine; MRI: magnetic resonance imaging; OCT: optical coherence tomography; SCAD: spontaneous coronary artery dissection.

Table S1. Therapies at 12-month by randomized treatment group.

**Therapy**	**Stratified treatment** (n= 45)	**Standard of care** (n = 45*)	**p value**
SAPT	15 (33.3)	21 (46.6)	0.197
DAPT	13 (28.9)	13 (28.9)	1
Beta-blockers	19 (42.2)	32 (71.1)	**0.006**
CCB	15 (33.3)	11 (24.4)	0.352
Non-dihydropyridine	14 (31.1)	0 (0.0)	**<0.001**
Dihydropyridine	4 (4.4)	11 (24.4)	0.065
ARBs/ACEi	22 (48.9)	30 (66.6)	0.088
Statins	37 (82.2)	35 (77.7)	0.598
Nitrates	2 (4.4)	1 (2.2)	0.500
Anticoagulant	7 (15.5)	12 (26.6)	0.197

Values are n (%).
Abbreviations: ACEi: Angiotensin-Converting Enzyme Inhibitors; ARBs: Angiotensin II Receptor Blockers; CCB: Calcium Channel Blockers; DAPT: Dual Antiplatelet Therapy; SAPT: Single Antiplatelet Therapy.
*two patients died at follow up before 12-month.

Table S2. Therapies at 12-month follow-up according to MINOCA aetiology in the stratified treatment group.

Therapy	Atherosclerotic Plaque Instability (n= 10)	Epicardial spasm (n= 16)	Microvascular spasm (n= 2)	Coronary embolism (n= 2)	SCAD (n= 6)	Undefined (n = 9)
SAPT	0 (0)	8 (50.0)	0 (0.0)	0 (0.0)	3 (50.0)	4 (44.4)
DAPT	8 (80.0)	0 (0.0)	0 (0.0)	0 (0.0)	3 (50.0)	2 (22.2)
Beta-blockers	5 (50.0)	0 (0.0)	1 (50.0)	1 (50.0)	5 (83.3)	6 (66.6)
CCB	1 (10.0)	13 (81.2)	0 (0.0)	0 (0.0)	1 (16.7)	0 (0.0)
ARBs/ACEi	5 (50.0)	6 (37.5)	1 (50.0)	1 (50.0)	4 (66.7)	5 (55.6)
Statins	7 (70.0)	13 (81.3)	2 (100.0)	2 (100.0)	6 (100.0)	7 (77.7)
Nitrates	0 (0.0)	1 (6.2)	0 (0.0)	0 (0.0)	0 (0.0)	1 (11.1)
Anticoagulant	1 (10.0)	1 (0.0)	0 (0.0)	2 (100.0)	0 (0.0)	3 (33.3)

Values are n (%).
Abbreviations: ACEi: Angiotensin-Converting Enzyme Inhibitors; ARBs: Angiotensin II Receptor Blockers; CCB: Calcium Channel Blockers; DAPT: Dual Antiplatelet Therapy; SAPT: Single Antiplatelet Therapy.

Table S3. Advanced diagnostic work-up for each patient enrolled in the stratified treatment group.

Patient	OCT performed	ACh performed	CMR performed	TEE performed	Diagnosis
1	✅	✅	✅	❌	Epicardial spasm
2	✅	✅	✅	❌	Undefined
3	✅	✅	✅	❌	Epicardial spasm
4	✅	✅	✅	❌	Epicardial spasm
5	✅	✅	❌	❌	Unstable Plaque
6	✅	✅	❌	❌	Unstable Plaque
7	✅	✅	✅	❌	Unstable Plaque
8	✅	✅	✅	❌	Epicardial spasm
9	✅	✅	✅	❌	Epicardial spasm
10	✅	✅	✅	❌	Epicardial spasm
11	✅	❌	❌	❌	SCAD
12	✅	✅	✅	❌	Epicardial spasm
13	✅	✅	❌	❌	Unstable Plaque
14	✅	✅	✅	❌	Unstable Plaque
15	✅	✅	✅	✅	Undefined
16	❌	✅	✅	❌	Epicardial spasm
17	✅	✅	❌	❌	Epicardial spasm
18	✅	✅	✅	❌	Unstable Plaque
19	✅	❌	✅	❌	Unstable Plaque
20	❌	✅	✅	❌	Epicardial spasm
21	✅	✅	✅	❌	Undefined
22	✅	✅	✅	❌	Undefined
23	✅	✅	✅	✅	Embolism
24	✅	✅	✅	❌	Undefined
25	✅	❌	✅	❌	SCAD
26	✅	✅	✅	❌	Epicardial spasm
27	✅	❌	✅	❌	SCAD
28	✅	❌	✅	❌	SCAD
29	✅	❌	✅	✅	Embolism
30	✅	✅	✅	❌	Undefined
31	✅	✅	✅	❌	Epicardial spasm
32	✅	✅	❌	❌	Unstable Plaque
33	✅	✅	✅	❌	Undefined
34	✅	✅	✅	❌	Epicardial spasm
35	✅	✅	❌	❌	Epicardial spasm
36	✅	✅	❌	❌	Unstable Plaque
37	✅	❌	✅	❌	SCAD
38	✅	✅	✅	❌	Undefined
39	✅	❌	✅	❌	SCAD
40	✅	✅	✅	❌	Unstable Plaque

41	✅	✅	✅	❌	Undefined
42	✅	✅	❌	❌	Epicardial spasm
43	✅	✅	✅	❌	Microvasc spasm
44	✅	✅	✅	❌	Epicardial spasm
45	✅	✅	✅	❌	Microvasc spasm

Legend: SCAD: spontaneous coronary artery dissection.
OCT was not performed in two patients due to extremely tortuous coronary vessels and because of the absence of suspicion of unstable plaque.
ACh was not performed in 8 patients due to an OCT diagnosis of SCAD (6 patients) or unstable plaque (1 patient) or coronary embolism (1 patient).
TEE was performed only if there were predisposing factors to coronary embolism (PFO, atrial fibrillation, prosthetic heart valves).
